# Dopamine neuron degeneration in the Ventral Tegmental Area causes hippocampal hyperexcitability in experimental Alzheimer’s Disease

**DOI:** 10.1038/s41380-024-02408-9

**Published:** 2024-01-16

**Authors:** Elena Spoleti, Livia La Barbera, Emma Cauzzi, Maria Luisa De Paolis, Luana Saba, Ramona Marino, Giuseppe Sciamanna, Vincenzo Di Lazzaro, Flavio Keller, Annalisa Nobili, Paraskevi Krashia, Marcello D’Amelio

**Affiliations:** 1 Department of Medicine and Surgery, Università Campus Bio-Medico di Roma, 00128 Rome, Italy; 2 Department of Experimental Neurosciences, IRCCS Santa Lucia Foundation, 00143 Rome, Italy; 3 Department of Systems Medicine, University of Rome Tor Vergata, 00133 Rome, Italy; 4 UniCamillus International University of Health Sciences, 00131 Rome, Italy; 5 Operative Research Unit of Neurology, Fondazione Policlinico Universitario Campus Bio-Medico, 00128 Rome, Italy; 6 Department of Sciences and Technologies for Sustainable Development and One Health, Università Campus Bio-Medico di Roma, 00128 Rome, Italy

**Keywords:** Neuroscience, Physiology

## Abstract

Early and progressive dysfunctions of the dopaminergic system from the Ventral Tegmental Area (VTA) have been described in Alzheimer’s Disease (AD). During the long pre-symptomatic phase, alterations in the function of Parvalbumin interneurons (PV-INs) are also observed, resulting in cortical hyperexcitability represented by subclinical epilepsy and aberrant gamma-oscillations. However, it is unknown whether the dopaminergic deficits contribute to brain hyperexcitability in AD. Here, using the Tg2576 mouse model of AD, we prove that reduced hippocampal dopaminergic innervation, due to VTA dopamine neuron degeneration, impairs PV-IN firing and gamma-waves, weakens the inhibition of pyramidal neurons and induces hippocampal hyperexcitability via lower D2-receptor-mediated activation of the CREB-pathway. These alterations coincide with reduced PV-IN numbers and Perineuronal Net density. Importantly, L-DOPA and the selective D2-receptor agonist quinpirole rescue *p*-CREB levels and improve the PV-IN-mediated inhibition, thus reducing hyperexcitability. Moreover, similarly to quinpirole, sumanirole – another D2-receptor agonist and a known anticonvulsant – not only increases *p*-CREB levels in PV-INs but also restores gamma-oscillations in Tg2576 mice. Conversely, blocking the dopaminergic transmission with sulpiride (a D2-like receptor antagonist) in WT mice reduces *p*-CREB levels in PV-INs, mimicking what occurs in Tg2576. Overall, these findings support the hypothesis that the VTA dopaminergic system integrity plays a key role in hippocampal PV-IN function and survival, disclosing a relevant contribution of the reduced dopaminergic tone to aberrant gamma-waves, hippocampal hyperexcitability and epileptiform activity in early AD.

## Introduction

Alzheimer’s Disease (AD) represents the main cause of cognitive impairment and dementia in the elderly, affecting over 55 million people worldwide according to the latest *World Alzheimer Report* [1].

The disease is progressively heralded by a cluster of symptoms as memory loss, language disturbances, psychological and psychiatric changes and difficulty in carrying out daily activities [2, 3]. Instead, the neuropathological hallmarks – intracellular neurofibrillary tangles, extracellular amyloid-β (Aβ) peptides, neuritic plaques and neuroinflammation – are based on autopsy studies from brains in the advanced disease stage.

One of the most sneaking aspects of AD is that the initial symptoms are preceded by a long pre-symptomatic stage during which the disease hallmarks are already operative in destroying synapses and connections, leading to derangement of electrical cerebral activity (ECA) and brain seizures [4–9]. Accordingly, clinical and epidemiological studies show ECA abnormalities and increased incidence of epileptic-like activity in patients, manifesting even before amnestic Mild Cognitive Impairment (aMCI) and clinical dementia [10, 11]. Network alterations underlying these seizures – including neuronal hyperexcitability – are common features in prodromal AD. Importantly, several evidence highlight that brain hyperexcitability per-se exacerbates pathophysiological processes, including Aβ accumulation, cognitive decline and the occurrence of dementia [11, 12]. In fact, aberrant neuronal activity enhances the generation of Aβ oligomers that contribute further to network desynchronization [13, 14]. Accordingly, aberrant brain networks are the ones most affected by Aβ accumulation, hyperphosphorylated tau or brain atrophy [7, 9], suggesting a loop in which early hyperexcitability might amplify the synaptic Aβ release, accelerating the disease progression [9, 15–19]. Cerebral cortex hyperexcitability in absence of atrophy or structural changes has been demonstrated also in AD patients using combined imaging and non-invasive stimulation techniques [20–22].

Brain hyperexcitability in AD is particularly associated with aberrant gamma-waves (30–120 Hz). These rhythms received attention because of their relationship to information processing and cognitive functions [23–26]. Gamma-oscillations are generated by the synchronous activity of GABAergic fast-spiking Parvalbumin interneurons (PV-INs), that modulate the balance between excitatory and inhibitory synaptic transmission, generating a window for a precise synchronization of pyramidal neurons (PNs) [23, 25, 27–31]. Many works claim PV-IN alterations and defective gamma-oscillations in AD patients and animal models, leading to network hyperexcitability [5, 7, 11, 19, 23, 28, 30, 32–37]. Indeed, rescuing the PV-IN activity or restoring gamma-waves ameliorates AD pathology in mice [18, 34, 38–41], paving the way for promising non-invasive treatments in patients [42–44].

Although mechanisms underlying PV-IN deficits remain elusive, some works suggest that dopamine directly increases inhibitory and/or oscillatory activity mediated by cortical and subcortical PV-IN firing [45–51]. This raises the hypothesis that deficits in the dopaminergic innervation onto PV-INs affect the functioning/firing of these neurons and worsen their oscillatory action. In this regard, recent clinical works show important alterations of the mesocorticolimbic dopamine system that include Ventral Tegmental Area (VTA) volume reduction, low functional VTA connectivity, hypometabolism, atrophy and neuroinflammation of mesocorticolimbic targets [52–61], all identified as predictive hallmarks of the pathology and of early conversion from MCI to AD [56, 59, 61, 62]. These clinical investigations stemmed from studies on the Tg2576 AD mouse model, where we previously revealed degeneration of VTA dopamine neurons in a pre-plaque stage [63–65]. This early event leads to reduced dopamine in projection areas, with consequent circuit impairments, memory and reward deficits [63–67]. Other AD models show similar data [68–70]. Importantly, reducing the VTA degenerative process [63, 71] or boosting the dopaminergic tone with various dopaminergic drugs can rescue these alterations in mice [64, 66, 72–74] and improve symptoms in patients [75–77].

Given these compelling evidence of precocious dopaminergic system dysfunctions in AD, our aim was to investigate whether the lower dopamine outflow in the Tg2576 hippocampus might result in network hyperexcitability driven by defects in PV-IN-mediated neurotransmission.

Here, we demonstrate that the Tg2576 hippocampus is characterized by decreased gamma-oscillations and increased brain excitability. These deficits are due to reduced PV-IN excitability and decreased GABAergic drive onto CA1 PNs. We also found a progressive reduction of PV-INs since 3 months of age, and significant alterations in the Perineuronal Nets (PNNs), a specialized extracellular matrix surrounding PV-INs, implicated in neuroprotection. Of note, Tg2576 PV-INs are characterized by lower expression of nuclear *p*-CREB – a transcriptional factor downstream of D2-like receptors – that regulates the expression of genes involved in survival and neuronal activity, including c-Fos, which was also reduced. Importantly, these events are related to reduced dopaminergic contacts onto PV-INs, since in-vivo and in-vitro dopaminergic treatments in Tg2576 mice enhance PV-IN *p*-CREB and c-Fos levels, restore GABAergic activity, and ameliorate hyperexcitability. Intriguingly, the D2-like receptor agonist sumanirole – known to have anticonvulsant activity against frontal lobe onset seizures [47] – restores *p*-CREB levels in PV-INs and rescues gamma-oscillations.

Overall, in this work we provide novel insights into the insurgence of early hippocampal hyperexcitability in AD, suggesting that the precocious VTA dopamine neuron degeneration, and the consequent dopamine loss, trigger defects in GABAergic transmission and brain oscillations, leading to network hyperexcitability and to epileptic seizures that characterize the disease.

## Materials and methods

### Animals

Heterozygous Tg2576 [78] and WT littermates of either sex were used at 1, 3 and 7 months of age. PV-Cre (JAX #017320) and DAT-Cre^IRES^ mice (JAX #006660) were crossed with Tg2576 males to generate PV-Cre/Tg2576 and DAT-Cre/Tg2576 mice, respectively. Animals were housed with *ad libitum* food and water, with a 12 h light/dark cycle.

In-vivo L-DOPA treatments (10 mg/kg) were performed as in [64, 66]. 3-month-old Tg2576 mice were perfused 1 h after the intraperitoneal (i.p.) injection of sumanirole (5 mg/kg) or saline (adapted from [47]). All experiments complied with the ARRIVE guidelines and were carried out according to ethical guidelines of the European Council (2010/63/EU). Experimental approval was obtained from the Italian Ministry of Health.

### Stereotaxic injections

6-month-old PV-Cre/WT and PV-Cre/Tg2576 animals were injected in the dorsal CA1 (0.2 µL/hemisphere; 20 nL/min; AP: −1.9; ML: +/−1.3; DV: −1.5) with the Cre-inducible AAV5-EF1a-DIO-mCherry (7.62 ×10^13^ particles/mL; gift from Karl Deisseroth, UNC Vector Core). DAT-Cre/WT and DAT-Cre/Tg2576 mice were injected in the left VTA (0.5 µL; 50 nL/min; AP: −3.2; ML: −0.35; DV: −4.4) with AAV1-hSyn-FLEx-mGFP-2ASyPhy-mRuby (gift from Liqun Luo; Addgene #71760-AAV1).

Mice were anaesthetized with Rompun (20 mg/mL, 0.5 mL/kg; Bayer) and Zoletil (100 mg/mL, 0.5 mL/kg; Virbac; i.p.) and positioned in a stereotaxic apparatus, before a burr hole was made under aseptic conditions. Viral vectors were infused via 1 μL Hamilton syringe (Neuros7001; #65458-01) using Pump11 Elite Nanomite (Harvard Apparatus). To ensure viral expression, mice were used 21–30 days following injection.

### Brain slicing and treatment with quinpirole or sulpiride

Slicing was performed as described previously [63, 65, 67]. Briefly, following halothane anesthesia, mice were transcardially perfused, decapitated and 300 μm parasagittal hippocampal slices were cut with Leica VT1200 vibratome in ice-cold oxygenated (95% O_2_, 5% CO_2_) solution (in mM: 92 NMDG, 2.5 KCl, 1.2 NaH_2_PO_4_, 30 NaHCO_3_, 20 HEPES, 25 Glucose, 5 Na-Ascorbate, 2 Thiourea, 3 Na-Pyruvate, 10 MgSO_4_, 0.5 CaCl_2_, ~295 mOsm; pH = 7.3–7.4). Then, slices were allowed to recover before recordings as in [63, 67].

For gamma-waves, horizontal hippocampal slices were cut in sucrose-based solution (in mM: KCl 3, NaH_2_PO_4_ 1.25, NaHCO_3_ 26, MgSO_4_ 10, CaCl_2_ 0.5, Glucose 25, sucrose 185; ~300 mOsm, pH = 7.4) and were allowed to recover for 1 h before recordings.

For ex-vivo quinpirole or sulpiride treatment, slices were incubated for 10 min in oxygenated artificial Cerebro-Spinal Fluid (aCSF, in mM: 124 NaCl, 1.25 NaH_2_PO_4_-H_2_O, 26 NaHCO_3_, 3 KCl, 10 Glucose, 1 MgSO_4_, 2 CaCl_2_, pH = 7.3–7.4). Drugs (μM): (-)-quinpirole hydrochloride (60, Tocris), (RS)-(±)-Sulpiride (100, Sigma), NBQX (10), AP-V (50), lidocaine hydrochloride (500, Abcam). Slices were then processed for immunofluorescence.

### Electrophysiological recordings

For whole-cell and gamma-wave recordings, a single hippocampal slice was placed under an upright microscope (BX51WI Olympus) and continuously perfused with oxygenated aCSF (30–32 °C; 3–4 mL/min). Recordings were performed using a MultiClamp-700B Amplifier, digitized with Digidata-1550B and computer-saved with pClamp11 (Molecular Devices; Fluo Opto Patch electrophysiology integrated system by Crisel Instruments). Patch-pipettes (<3 MΩ for field recordings; 3–5 MΩ for patch-clamp) were pulled from borosilicate capillaries (TW-150F-4, WPI). Analysis was performed with Clampfit11.

For field Excitatory Post Synaptic Potentials (fEPSPs) and Population spikes (POPs), the hippocampal slice was perfused with aCSF containing 0.5 mM L-glutamine (30–32 °C; 10 mL/min); signals were obtained with a MED64 multielectrode-array device, acquired using the Mobius software, digitized at 20 kHz and low-pass filtered at 1 Hz (Alpha MED Sciences) [79].

#### Field recordings

Gap-free gamma-wave recordings were obtained from the CA1 *Stratum Pyramidale* following 10 min of bath-applied Carbachol (CCh, 20 μM, Abcam). The patch-pipette contained (in mM): 140 KMeSO_4_, 10 KCl, 10 HEPES, 2 Mg_2_ATP, 0.4 Na_3_GTP; pH = 7.25 with KOH and was placed in the slice at a 65°-angle. Voltage-clamp signals were low-pass filtered at 3 kHz, digitized at 10 kHz and post-hoc filtered at 1 kHz. Frequency components were evaluated from power spectra, constructed for 60 s epochs, using a fast Fourier transform. The integrated power of 20–60 Hz was used to quantify the gamma-oscillation power. The peak frequency was the frequency at which the power spectrum peak occurred in the gamma-range.

The effect of sumanirole (10 μM; Tocris) on gamma-waves was evaluated following bath application.

fEPSPs and POPs were recorded from the CA1 *Stratum Radiatum* or *Pyramidale*, respectively, following 200 μs Schaffer-collateral stimulation. Input/Output (I/O) curves were obtained by measuring the fEPSP slope or the principal Population spike (PS1) amplitude at 20 μA-stepped afferent stimulation, every 30 s.

For POPs, after 3 min stable responses at half-maximum stimulation, (−)-Bicuculline methochloride (5 μM) was bath-applied for 10 min to elicit epileptiform activity. The averaged responses were used for calculating the POP number (amplitude-threshold = 0.02 mV), the total response duration (from onset of the primary spike to the offset of the last spike), the PS1 amplitude and duration and the maximum peak [79, 80].

#### Patch-clamp recordings

Spontaneous Inhibitory Post-Synaptic Currents (sIPSCs) were recorded from CA1 PNs at -70 mV in aCSF containing (μM): NBQX (10), AP-V (50) and CGP-55845 (1). Patch-pipettes contained (in mM): 140 CsCl, 1 MgCl_2_, 10 HEPES, 4 Mg_2_-ATP, 2.5 QX-314 Chloride; pH = 7.29 with CsOH; 295 mOsm. Voltage signals were digitized at 20 kHz and low-pass filtered at 4 kHz. Data were collected after 3 min wash-on of inhibitors and after 6-min of L-DOPA application to ensure steady-state. Experiments in which access resistance exceeded 25% were discarded. No liquid junction-potential correction was applied.

sIPSCs were detected with the threshold-crossing function of pClamp11 from three different 30-s-long analysis-windows/cell. Events were analyzed for amplitude (I_peak_), instantaneous frequency (Inst. Freq.) and total charge transfer (pA*ms). Parameters were tested for time stability using the Pearson’s correlation test; segments of events showing time instability were excluded.

The effect of L-DOPA (10 μM, Tocris) on sIPSCs was evaluated following bath application.

CA1 fast-spiking PV-INs were identified by mCherry-positivity following excitation at ~525 nm (Lambda421 Led Illumination system from Sutter Instruments, through MetaFluor-software from Molecular Devices). Current-clamp recordings were acquired at 50 kHz, low-pass filtered at 10 kHz. Patch-pipettes contained (in mM): 120 potassium-gluconate, 20 KCl, 10 HEPES, 0.2 EGTA, 4 Mg_2_-ATP, 0.3 Na_2_-GTP, 10 Na-Phosphocreatine, 2 MgCl_2_; pH = 7.3, 295 mOsm. Membrane capacitance (C_m_) and resistance (R_m_) were estimated using the amplifier inbuilt voltmeter immediately after gaining cell access. 1-min-long recording (I = 0) was performed to estimate the Resting Membrane Potential (RMP). Then, PV-INs were kept at −60 mV by direct current injection and we applied a 1-s-long protocol (50 pA-stepped, 15 s apart, from −200 pA to +700 pA) to evaluate sub- and supra-threshold properties. AP detection was performed with a + 5 mV-threshold crossing method to build *f*-I curves; input resistance (R_in_), sag amplitude, SFA and CV-ISI were evaluated as in [81]. The AP threshold and rheobase were estimated by 50 ms-long steps (5 pA; from 0 to +400 pA) as in [67].

### Immunofluorescence

Mice were anaesthetized with Rompun/Zoletil and perfused transcardially with saline followed by 4% PFA in Phosphate Buffer (PB; 0.1 M, pH = 7.4). Brains or treated slices (see above) were fixed in PFA and immersed in 30% sucrose until sinking (4 °C).

30 μm-thick sections were cut using a cryostat, slices were collected in PB and processed with primary antibodies; after three washes in PB slices were incubated with secondary antibodies for 2 h (RT). When necessary, DAPI was used to counterstain nuclei.

For PV^+^/PNN^+^ immunofluorescence, every second slice was processed alternatively with anti-PV and with Wisteria Floribunda Agglutinin (WFA) antibodies for three nights in PB containing 0.3% Triton X-100 (PB-Triton 0.3%; 4 °C). Quantification of 3D-reconstructed images was done on 12–15 cells/animal. The WFA intensity around individual PV-IN soma was analyzed by randomly drawing 10 regions of interest (ROI; 25 × 25 pixels). PV-IN area was analyzed on single plan images at nucleus focus level with Neurolucida software (2021 v, MicroBright-Field).

To validate the injection and expression of AAV1-hSyn-FLEx-mGFP-2ASyPhy-mRuby in dopamine neurons, VTA sections were stained overnight with anti-TH antibody in PB-Triton 0.3% (4 °C).

Dopaminergic synapses onto PV-IN soma were identified as mRuby^+^ puncta in slices from AAV1-hSyn-FLEx-mGFP-2ASyPhy-mRuby-injected mice, incubated for two nights with anti-PV and NeuroTrace antibodies in PB-Triton 0.3% (4 °C). The puncta fluorescence intensity was obtained as described below, analyzing 10–12 cells/animal. For the synaptic puncta density, a semi-automatic count was performed with ImageJ (http://imagej.nih.gov/ij/). Animals with mismatched injection were excluded.

For PV/*p*-CREB labeling, slices were incubated with anti-PV and anti-*p*-CREB antibodies in PB-Triton 0.3% for two nights at 4 °C. For PV/c-Fos labeling, slices were incubated with anti-PV and anti-c-Fos antibodies in PB-Triton 0.3% and 10% donkey serum for two nights at 4 °C. To analyze nuclear *p*-CREB and c-Fos levels, images from at least 3-4 slices were taken on single plan at nucleus focus level.

For NaV1.1/PV labeling, slices were incubated in 10 mM Sodium citrate buffer (containing 0.05% Triton X-100; 20 min, 75 °C, pH = 6), rinsed in PB, immersed in blocking solution (5% donkey serum, 0.2% Triton X-100 in PB) for 1 h and incubated with primary antibodies in blocking solution (overnight at 4 °C). To analyze NaV1.1 levels, images from at least 3-4 slices were taken on single plan at nucleus focus level.

For TH fiber immunofluorescence, sections were incubated in 10 mM Sodium citrate buffer (containing 0.05% Triton X-100; 20 min, 75 °C, pH = 6), rinsed in PB, immersed in blocking solution (5% donkey serum, 0.2% Triton X-100 in PB) for 1 h and incubated with primary antibody in blocking solution (overnight at 4 °C) [67]. For TH level quantification, 3D-images were collected from at least 4–5 slices processed simultaneously and analyzed by ImageJ, by randomly drawing 8 ROI (150 × 150 pixels). TH^+^ fiber density was quantified manually (number of fibers/250 µm). Data obtained from each region were averaged for slices, and these data were averaged per animal.

PV-IN specificity of AAV5-EF1a-DIO-mCherry was confirmed by co-labeling PV with mCherry [82].

#### Primary antibodies

c-Fos (1:200; Abcam; #AB7963; RRID: AB_306177); *p*-CREB (1:200, Sigma-Aldrich; #S-06-519; RRID: AB_310153); NaV1.1 (1:500, Sigma-Aldrich; #AB5204; RRID: AB_91751); PV (1:500, Millipore; #MAB1572; RRID: AB_2174013); TH (1:1000, Abcam; #AB112; RRID: AB_297840); WFA (1:500, Sigma-Aldrich; #L1516; RRID: AB_2620171).

#### Secondary antibodies

Alexa Fluor-488 donkey anti-mouse (1:200; ThermoFisher; #R37114; RRID: AB_2556542), Alexa Fluor-555 donkey anti-rabbit (1:200; ThermoFisher; #A31572; RRID: AB_162543), Streptavidin Alexa Fluor-488-conjugated (1:1000; ThermoFisher; #S32354; RRID: AB_2315383); NeuroTrace-640/660 (1:200; ThermoFisher; #N21483; RRID: AB_2572212).

Sections were coverslipped with Aqueous Mounting Media (Sigma-Aldrich) and examined under a confocal laser-scanning microscope (Nikon Eclipse Ti2). For 3D-reconstruction, images were collected as Z-stacks and processed by maximum intensity projection. The immunolabelling specificity was confirmed by use of normal serum instead of primary antibodies (negative controls). For each staining, samples were acquired with the same laser settings. For quantitative analysis, images were processed simultaneously and exported with ImageJ: the signal was quantified by outlining the ROI and measuring the relative fluorescence intensity, as mean fluorescence intensity (F) over a defined surface area (A).

### Stereology

Every second slice of dorsal hippocampus or VTA was processed to estimate PV^+^ and PV^+^/PNN^+^ INs or TH^+^ neurons, respectively [63, 65, 79, 83]. DAPI or TH staining was used to define hippocampal or VTA boundaries. We applied an optical fractionator stereological design using the StereoInvestigator System (2021 v, MicroBright-Field). A stack of MAC5000 controller modules (Ludl Electronic Products) was interfaced with a Zeiss AxioImager KMAT with a motorized stage and an Axiocam506 camera. A 3D-optical fractionator counting probe (x, y, z of 90 × 90 × 25 μm for hippocampus and 50 × 50 × 25 μm for VTA) was applied. The ROI was outlined using the 5× objective; PV-INs and TH^+^ neurons were marked with a 40× or 100×-oil objective, respectively. Cell numbers were estimated according to Equation1:

1 $${{{{{\rm{N}}}}}}={{{{{\rm{SQ}}}}}}\times (1/{{{{{\rm{ssf}}}}}})\times (1/{{{{{\rm{asf}}}}}})\times (1/{{{{{\rm{tsf}}}}}})$$

where SQ represents neuron numbers in all optically sampled fields, ssf is the section sampling fraction, asf is the area sampling fraction and tsf is the thickness sampling fraction.

### Sample size, randomization, blinding

Sample numbers were determined with power analysis using the G*Power software (3.1.9.7 v) with input values of: power 0.8, errors 0.05 and standard deviations of WT and Tg2576 obtained from our previous publications.

Randomization (i.e. how each mouse from the same litter was ‘destined’ for each experimental group) was performed with a random number table, matched by sex and age.

Researchers were blinded to the genotype/treatment of each animal; un-blinding occurred after data analysis.

The experimental units are described in Figure legends. Briefly: neurons/slices for electrophysiology, mice for stereology and microscopy, and neurons for synaptic puncta analysis, c-Fos and *p*-CREB analysis following quinpirole and sulpiride treatment.

### Statistical analysis

Analysis was performed using GraphPad Prism (v8.01). When 2 independent factors were examined (age and genotype), data were analyzed by Two-Way ANOVA, followed by Tukey’s, Bonferroni’s or Sidak’s post-hocs. When no interaction was observed between independent parameters, we used *t*-tests. Data were checked for normality using D’Agostino–Pearson or Shapiro–Wilk tests; data sets were then analyzed accordingly with two-tailed parametric or non-parametric tests. The effects of L-DOPA on sIPSCs and sumanirole on gamma-oscillations were analyzed with Wilcoxon paired test or simple paired *t*-test, respectively. *f*-I, I/O curves and the Bicuculline effect were examined using two-way Repeated Measures ANOVA (with genotype and injected current/stimulus intensity/time as independent factors, respectively). The effect of quinpirole on *p*-CREB and c-Fos was analyzed with one-way ANOVA.

See Figure legends for more details. *p* ≤ 0.05 indicates statistical significance. In box-and-whisker plots the center line denotes the median, edges are upper/lower quartiles, whiskers show minimum/maximum values; points are individual experiments. All other data are presented as mean ± s.e.m.

## Results

### Impaired hippocampal gamma-oscillations and PV-IN firing in Tg2576 mice

**Fig. 1:**
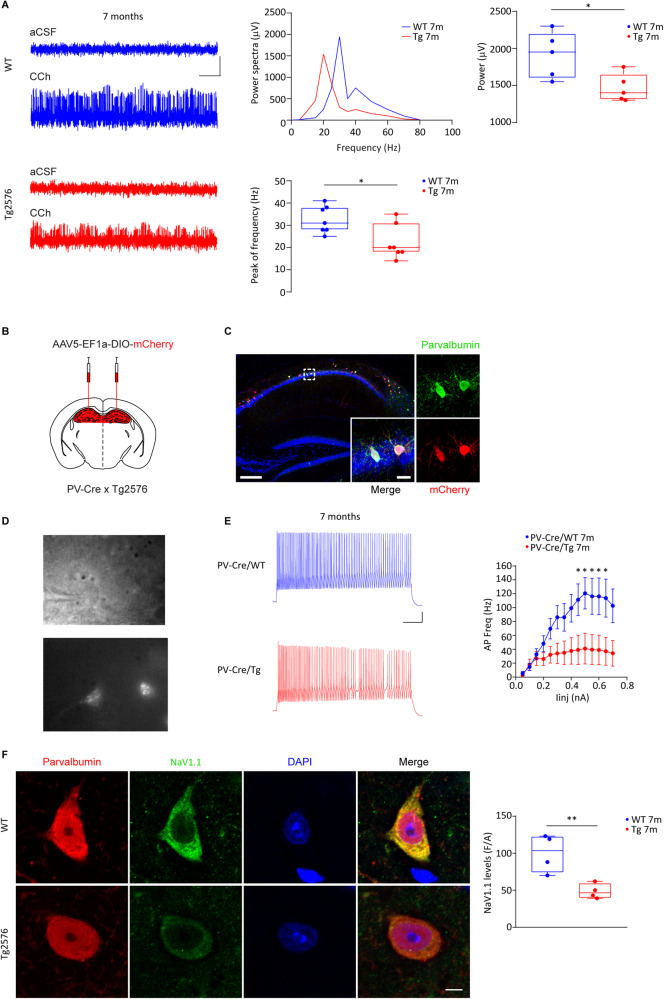
Gamma oscillations and PV-IN firing are altered in Tg2576 mice. **A** Representative traces of local field potentials recorded in the hippocampal pyramidal layer of 7-month-old WT and Tg2576 mice before (aCSF) and during CCh bath-perfusion (scale bars: 500 ms, 200 μV). (*Upper*) The representative power spectra and relative plot for these signals acquired during CCh perfusion indicate a reduced gamma-power in Tg2576 mice compared to WTs; (*bottom*) the plot shows the peak frequency reduction in the gamma-frequency range in Tg2576 mice with respect to WT littermates (*n* = 7 slices, 3 WT and Tg2576 mice; power: *p = 0.031; peak frequency: *p = 0.017, all with unpaired *t*-test with Welch’s correction). **B** Schematic representation of AAV5-EF1a-DIO-mCherry stereotaxic bilateral injections in the dorsal hippocampus of PV-Cre/Tg2576 and PV-Cre/WT mice. **C** Representative confocal images showing the selective viral infection (mCherry, red) of PV-INs (PV, green; scale bar: 200 μm). Inserts show high magnification of infected cells (scale bar: 20 μm). **D** Images show patch-clamp recordings from a CA1 PV-IN visualized with infrared videomicroscopy (*top*) and fluorescence (*bottom*). **E** Example traces show action potentials fired by PV-INs from 7-month-old PV-Cre/WT and PV-Cre/Tg2576 mice following 50 pA stepped current injection (scale bars: 12 mV; 125 ms). The *f*-I curves (±s.e.m.) show a reduced number of action potentials recorded from CA1 PV-INs of PV-Cre/Tg2576 mice compared to PV-Cre/WTs (*n* = 11 cells, 4 PV-Cre/WT mice; *n* = 6 cells, 4 PV-Cre/Tg2576 mice; two-way RM ANOVA, with Bonferroni’s multiple comparisons post-hoc test, genotype x injected current, *F*_13,182_ = 3.001, ***p = 0.0005; WT *vs* Tg2576: *p = 0.046, 0.45 nA, *p = 0.018, 0.50 nA; *p = 0.026, 0.55 nA; *p = 0.025, 0.60 nA; *p = 0.027, 0.65 nA). **F** Representative confocal images of NaV1.1 (green) in labeled CA1 PV-INs (PV, red) in 7-month-old WT and Tg2576 mice (scale bar: 5 µm). NaV1.1 levels are reduced in Tg2576 PV-INs compared to WTs (*n* = 4 WT and Tg2576 mice; **p = 0.009 with unpaired *t*-test). The nuclei are visualized by DAPI (blue).

Hippocampal PV-IN-mediated gamma-oscillations are essential for information processing, memory storage and retrieval, sensory encoding and neuronal assembly formation [30, 37]. Since Tg2576 mice, at 7 months of age, show relevant hippocampal alterations [63, 64, 66, 78, 84, 85], we used mice at this age to explore whether the hippocampal rhythmic activity is altered (Fig. 1A). We performed recordings of CA1 gamma-oscillations induced by CCh, known to activate cholinergic receptors onto PV-INs [29, 86]. By power spectrum analysis we readily observed a reduction in the power and peak frequency of Tg2576 CCh-induced gamma-waves (Fig. 1A), proving an impairment in the hippocampal oscillatory circuit. Given this result, we focused our attention on PV-IN membrane properties and evoked excitability. To selectively identify PV-INs, we used PV-Cre mice crossed with Tg2576, and injected the PV-Cre/WT and PV-Cre/Tg2576 offspring with the AAV5-EF1a-DIO-mCherry; confocal microscopy proved the selectivity of mCherry for PV-INs (Fig. 1B–D).

To assess sub- and supra-threshold changes in PV-Cre/Tg2576 mice, we recorded voltage-responses to injected currents. We observed no changes in passive membrane properties, in AP threshold or in rheobase of Tg2576 PV-INs (Supplementary Table 1). Nonetheless, the analysis of *f*-I curves revealed an important rundown of Tg2576 PV-IN evoked excitability compared to age-matched WTs (Fig. 1E), without changes in the pattern and regularity of firing (Supplementary Table 1). Since AP generation and propagation depend on voltage-gated sodium channels, we measured the levels of NaV1.1, one of the most expressed sodium channels in GABAergic neurons [36, 87–89]. 7-month-old Tg2576 mice had reduced levels of NaV1.1 in PV-INs (Fig. 1F), accounting for the reduced excitability of PV-INs we observed. These results can explain the impaired Tg2576 hippocampal gamma-oscillations.

### The reduced GABAergic transmission in Tg2576 mice is linked to hippocampal hyperexcitability

**Fig. 2:**
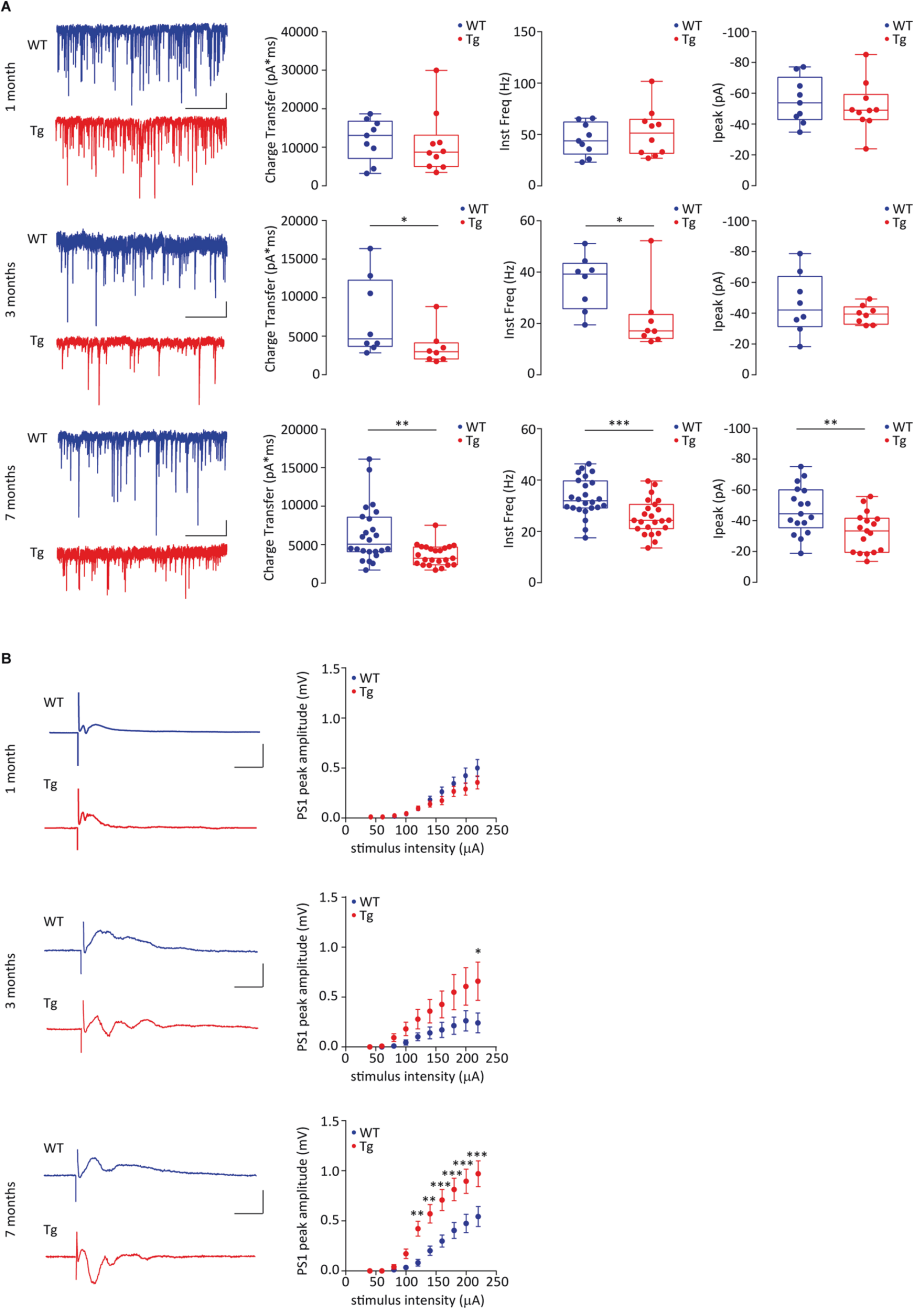
Tg2576 mice show reduced GABAergic transmission and hippocampal hyperexcitability starting at 3 months of age. **A** Examples of sIPSCs recorded from CA1 PNs of 1-, 3- and 7-month-old Tg2576 and WTs (scale bars: 25 pA; 2 s). (*Top*) Plots show no differences between genotypes at 1 month of age (*n* = 9 cells, 4 WT mice; *n* = 10 cells, 4 Tg2576 mice; Total charge transfer: *p* = 0.549 with Mann–Whitney U test; Inst. Freq.: *p* = 0.486 with unpaired *t*-test with Welch’s correction; I_peak_: *p* = 0.581 with unpaired *t*-test with Welch’s correction). (*Middle*) 3-month-old Tg2576 mice show reduced total charge transfer and Inst. Freq. of sIPSCs with respect to WTs, whilst no changes were detected in peak amplitude (*n* = 8 cells, 4 WT mice, *n* = 8 cells, 6 Tg2576 mice; Total charge transfer *p = 0.038; Inst. Freq. *p = 0.021, all with Mann–Whitney U test). (*Bottom*) 7-month-old Tg2576 mice show reduced total charge transfer, Inst. Freq. and peak amplitude of sIPSCs compared to age-matched controls (*n* = 24 cells, 6 WT mice; *n* = 23 cells, 6 Tg2576 mice; Total charge transfer **p = 0.006 with Mann–Whitney U test; Inst. Freq. ***p = 0.007 with unpaired *t*-test with Welch’s correction; I_peak_ **p = 0.006 with unpaired *t*-test with Welch’s correction). **B** Representative traces showing POPs recorded from the CA1 *stratum pyramidale* following half-maximal Schaffer collateral stimulation (scale bars: 0.25 mV; 5 ms; *Left*) and complete I/O curves (±s.e.m.; *Right*) in 1-, 3- and 7-month-old WT and Tg2576 mice. (*Top*) Plot shows similar I/O curves between genotypes at 1 month of age (*n* = 16 slices, 4 WT mice; *n* = 12 slices, 4 Tg2576 mice; two-way RM ANOVA with Bonferroni’s multiple comparison post-hoc test, genotype x stimulus intensity, *F*_9,234_ = 1.566, *p* = 0.126). (*Middle*) 3-month-old Tg2576 mice show increased POP peak amplitude in response to the maximum stimulation compared to controls (*n* = 7 slices, 4 WT mice; *n* = 8 slices, 6 Tg2576 mice; two-way RM ANOVA with Bonferroni’s, genotype x stimulus intensity, *F*_9,117_ = 2.610, *p* = 0.009; *p = 0.044 at 220 μA). (*Bottom*) I/O curves from 7-month-old Tg2576 mice show bigger POP peak amplitude than controls in response to increasing afferent stimulations (*n* = 14 slices, 6 WT mice; *n* = 12 slices, 6 Tg2576 mice; two-way RM ANOVA with Bonferroni’s, genotype x stimulus intensity, *F*_9, 216_ = 7.415, *p* < 0.0001; **p = 0.008, 120 μA, **p = 0.003, 140  μA, ***p = 0.0006, 160 μA, ***p = 0.0006, 180 μA, ***p = 0.0003, 200 μA, ***p = 0.0003, 220 μA).

Based on the reduced propensity of Tg2576 PV-INs to fire APs, and given the fact that these cells control the activity of hippocampal PNs, we next investigated whether the inhibitory transmission onto PNs is altered in Tg2576 mice. To this aim, we recorded sIPSCs from CA1 PNs of 1-, 3- and 7-month-old mice. Our data revealed that the sIPSC total charge transfer – the synaptic charge transferred by each IPSC – is reduced in 3- and 7-month-old Tg2576 compared to controls, indicating an impairment of the inhibitory drive onto PNs (Fig. 2A). Moreover, the sIPSC frequency was reduced in 3-month-old Tg2576 mice whilst the amplitude was similar between genotypes (Fig. 2A) and worsened in 7-month-old Tg2576 mice (Fig. 2A). Of note, the GABAergic transmission was unaltered between genotypes at 1 month of age (Fig. 2A).

These data prove that the GABAergic defects in 3- and 7-month-old Tg2576 mice are associated with reduced PV-IN activity. To investigate whether these perturbations contribute to a shift towards hyperexcitability in the Tg2576 hippocampus, we recorded POPs as a marker of brain hyperexcitability. As above, mice were used at 1, 3 and 7 months of age to directly link potential hyperexcitability to the progressive GABAergic defects. 3-month-old Tg2576 slices showed an increase in the PS1 peak amplitude only at maximum stimulation (Fig. 2B). Instead, 7-month-old Tg2576 PNs revealed a pronounced predisposition to hyperexcitability, evident by the increased PS1 amplitude at most stimulation intensities (Fig. 2B). On the other hand, 1-month-old Tg2576 slices did not show hyperexcitability with respect to WTs (Fig. 2B). This result verifies our hypothesis that a progressive reduction in the GABAergic drive onto PNs, starting after 1 month of age, induces hyperexcitability in the Tg2576 hippocampus.

A closer look at the Fig. 2B traces from 3- and 7-month-old Tg2576 indicates a tendency of PNs to evoke multiple POPs, although the analysis of the total duration, total peak number or PS1 duration at half-maximum stimulation did not show statistical significance (Supplementary Fig. 1A). To further strengthen this result, we bath-applied Bicuculline, a GABA_A_R antagonist, to induce epileptiform activity. As expected, Bicuculline elicited multiple POPs and increased the response duration in all slices (Supplementary Fig. 1B). Although no differences were detected between genotypes in 3-month-old animals, the Bicuculline-induced effect was significantly stronger in 7-month-old Tg2576 compared to WTs (Supplementary Fig. 1B), supporting the higher predisposition of Tg2576 PNs for epileptic-like activity.

Of note, the CA3-CA1 basal excitatory synaptic transmission was similar between genotypes across ages (Supplementary Fig. 2), as we previously reported [64, 66]. Overall, these results indicate a link between the defective PV-IN-mediated GABAergic transmission and the insurgence of brain hyperexcitability and epileptiform activity in the Tg2576 hippocampus.

### The number of hippocampal PV-INs is reduced in Tg2576 mice

**Fig. 3:**
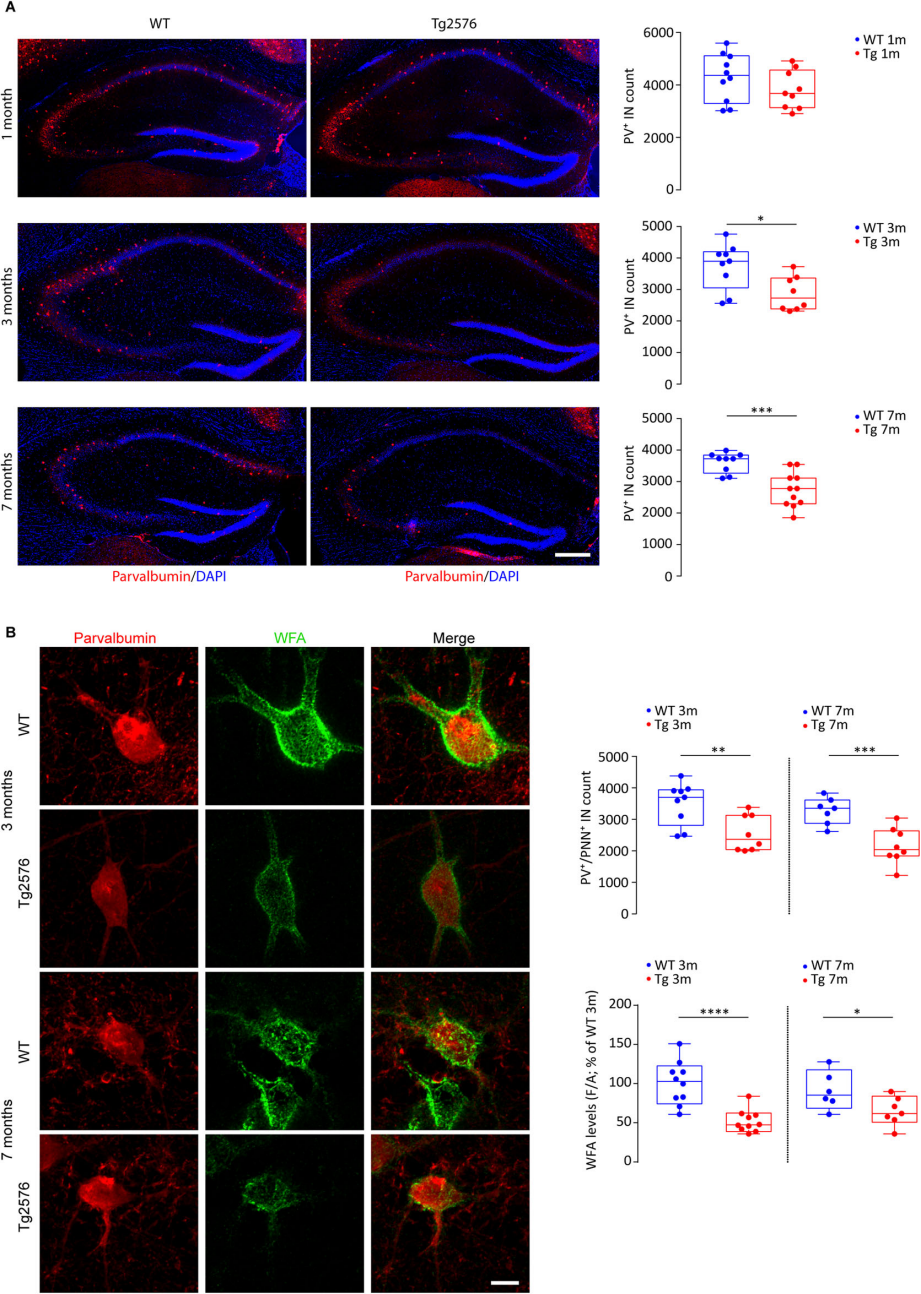
Reduced number of PV-INs and PNN alterations in Tg2576 mice since 3 months of age. **A** Representative confocal images of labeled PV-INs (PV, red) in coronal brain sections of dorsal hippocampus (DAPI, blue) of WT and Tg2576 mice at 1, 3 and 7 months of age and related stereological cell count (scale bar: 250 μm). The plot of 1-month-old mice indicates similar stereological cell counts for PV-INs between genotypes (*n* = 10 WT, *n* = 9 Tg2576 mice; *p* = 0.220); bottom plots show the reduction of PV-INs in the hippocampus of 3- and 7-month-old Tg2576 mice compared to WTs (*3-month-old*: *n* = 9 WT, *n* = 8 Tg2576 mice; *p = 0.015; *7-month-old*: *n* = 9 WT, *n* = 11 Tg2576 mice; ***p = 0.0005; all with unpaired *t*-test). **B** Analysis of confocal Z-stack double immunofluorescent labeling of PV^+^/PNN^+^ INs (PV, red; WFA, green) in the dorsal hippocampus of 3- and 7-month-old WT and Tg2576 mice (scale bar: 10 μm); (*top*) the stereological cell count plot shows the reduced number of Tg2576 PV^+^/PNN^+^ INs compared to age-matched controls at both ages (*3-month-old*: *n* = 9 WT, *n* = 8 Tg2576 mice; **p = 0.007; *7-month-old*: *n* = 7 WT, *n* = 8 Tg2576 mice; ***p = 0.001; all with unpaired *t*-test); (*bottom*) the plot shows reduced WFA fluorescence intensity in Tg2576 PV-INs at 3 and 7 months of age (*3-month-old*: *n* = 10 WT and Tg2576 mice; ****p < 0.0001; *7-month-old*: *n* = 6 WT and *n* = 7 Tg2576 mice; *p = 0.043; all with unpaired *t*-test).

We next asked whether these events might also reflect changes in the number of PV-INs and density of PNNs, the extracellular matrix that surrounds PV-INs ensuring synapse stabilization, GABA release and neuroprotection [90–93]. We focused on PNNs because of controversies regarding changes in abundance and distribution in different AD models [94]. We readily saw that PV-IN numbers are lower in 3- and 7-month-old Tg2576 (Fig. 3A) and similar results were obtained for PV-INs enwrapped by PNNs (PV^+^/PNN^+^; Fig. 3B). Furthermore, although the PV soma was similar between genotypes, indicating no cellular shrinkage (Supplementary Fig. 3A), the PNN density, analyzed by WFA fluorescence intensity, was lower in Tg2576 mice at both ages (Fig. 3B). Importantly, PV-IN and PV^+^/PNN^+^ cell numbers were unchanged in 1-month-old Tg2576 mice (Fig. 3A and Supplementary Fig. 3B), proving an age-dependent and progressive loss of PV-INs that we detected between 1- and 3-months.

### The VTA dopaminergic input onto Tg2576 hippocampal PV-INs is reduced

Our previous works revealed that Tg2576 mice are characterized by selective degeneration of VTA dopamine neurons since 2-3 months of age, that leads to lower hippocampal dopamine, as well as functional and behavioral deficits that can be rescued by boosting the dopaminergic tone [63, 64, 66, 67]. Thus, we pursued the possibility that the observed reduced GABAergic tone from PV-INs and the consequent hippocampal hyperexcitability in Tg2576 mice relate to the lower VTA dopaminergic input.

**Fig. 4:**
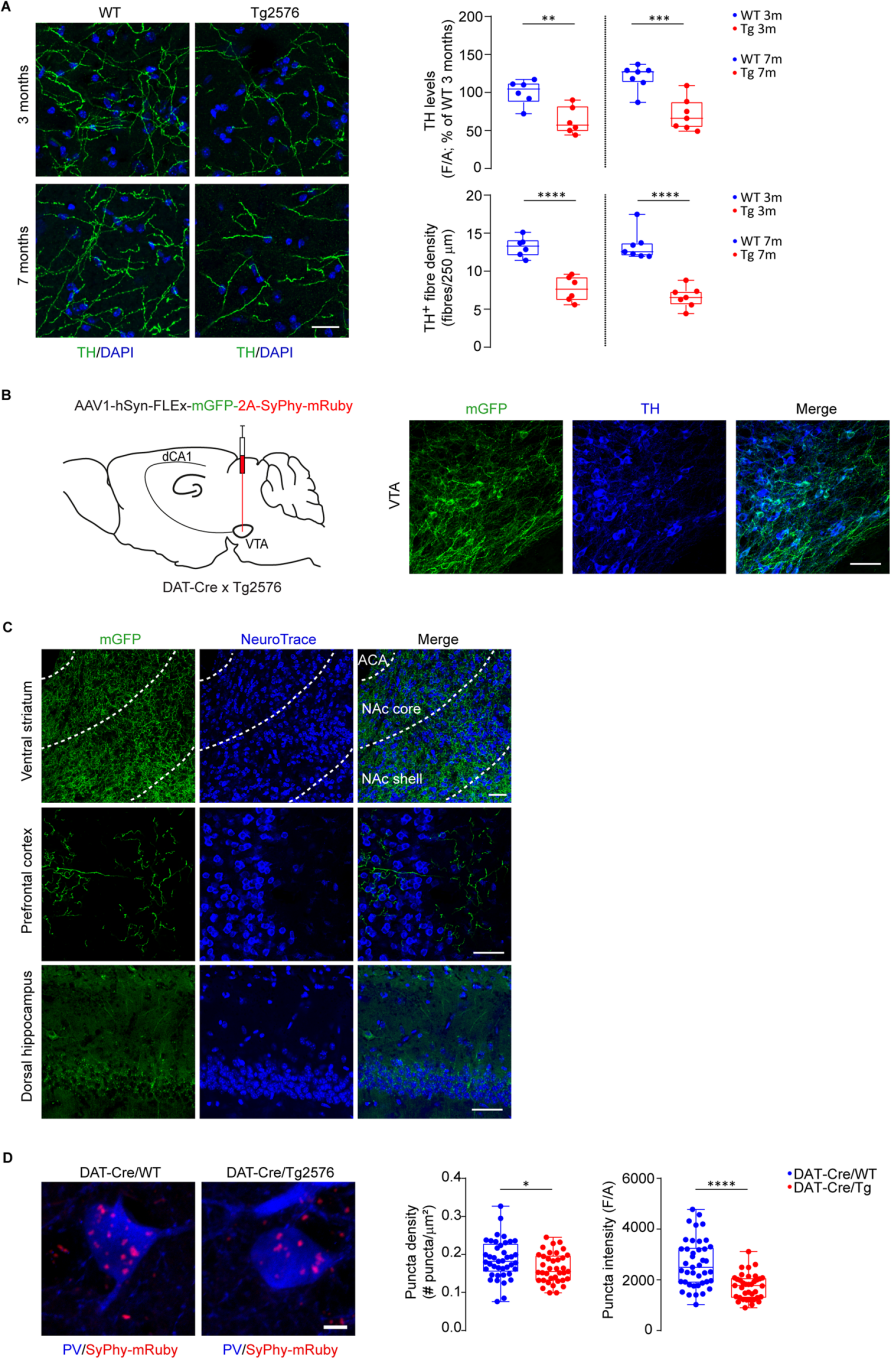
The dopaminergic input from the VTA is reduced in hippocampal PV-INs of Tg2576 mice. **A** Z-stack immunofluorescent labeling of TH^+^ fibers (green) of dorsal hippocampus (scale bar: 25 μm). The upper plot depicts densitometric values of TH (*3-month-old*: n = 6 WT and Tg2576 mice; **p = 0.004; *7-month-old*: *n* = 7 WT and Tg2576 mice; ***p = 0.0004; all with unpaired *t*-test) in 3- and 7-month-old WT and Tg2576 mice. The bottom plot shows mean TH^+^ fiber density (fibers/250 µm; *3-month-old*: *n* = 6 WT and Tg2576 mice; ****p < 0.0001; *7-month-old*: *n* = 7 WT and Tg2576 mice; ****p < 0.0001; all with unpaired *t*-test). **B** Schematic representation of AAV1-hSyn-FLEx-mGFP-2A-SyPhy-mRuby stereotaxic injection in the left VTA of DAT-Cre/Tg2576 and DAT-Cre/WT mice and representative confocal images showing the selective infection of VTA TH^+^ neurons (blue) with the injected AAV (mGFP, green; scale bar: 50 μm). **C** Representative confocal images (scale bar: 50 μm) showing 3 different VTA projection areas (NeuroTrace, blue) containing mGFP-labeling (Ventral striatum, Prefrontal cortex and the dorsal hippocampus; ACA: Anterior Cerebral Artery). **D** Representative z-stack confocal images showing the density of dopamine synapses (mRuby, red) onto CA1 PV-INs (blue) and relative plots showing reduced dopaminergic synapse density (*left*) and mean fluorescence (*right*) in CA1 PV-INs of 7-month-old Tg2576 mice compared to age-matched WTs (*n* = 41 cells, 3 WT mice; *n* = 37 cells, 3 Tg2576 mice; *density* as #puncta/μm^2^: *p = 0.028 with unpaired *t*-test; *mean fluorescence*: ****p < 0.0001; unpaired *t*-test with Welch’s correction; scale bar: 10 μm).

We confirmed that tyrosine hydroxylase (TH, the enzyme for dopamine production) levels and the TH^+^ fiber density are lower in the dorsal hippocampus of Tg2576 mice both at 3 and at 7 months of age (Fig. 4A). To prove the reduced dopamine input onto PV-INs, we crossed DAT-Cre with Tg2576 mice and injected the offspring with the AAV1-hSyn-FLEx-mGFP-2ASyPhy-mRuby virus in the VTA. This AAV allows the labeling of the dopamine cell soma and processes with mGFP and of the presynaptic (synaptophysin-containing) sites with mRuby. With confocal microscopy we proved the co-labeling between TH^+^-cell bodies and mGFP in the VTA (Fig. 4B) and we also confirmed the presence of mGFP-labeled terminals in the ventral striatum and medial prefrontal cortex, the main VTA dopamine projecting areas (Fig. 4C). mGFP-labeled dopaminergic processes – albeit scarce – were also observed in the hippocampus (Fig. 4C). Importantly, we found a reduction in both mRuby^+^ density and fluorescence intensity on hippocampal PV-INs from 7-month-old DAT-Cre/Tg2576 mice (Fig. 4D), indicating a reduction in dopaminergic contacts onto these neurons. This is in line with the reduced number of TH^+^ neurons in the VTA of DAT-Cre/Tg2576 mice (Supplementary Fig. 3C).

**Fig. 5:**
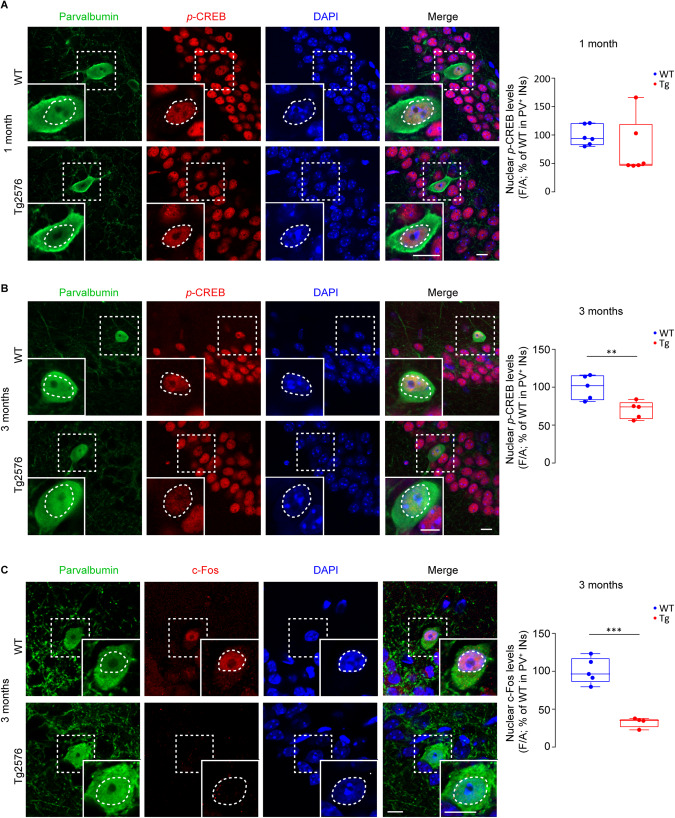
Reduced *p*-CREB and c-Fos levels in PV-INs of 3-month-old Tg2576 mice. **A** Confocal images of nuclear *p*-CREB (red) in CA1 PV-INs (green) of 1-month-old Tg2576 and WT mice (scale bar: 10 µm each). The plot shows no changes in nuclear *p*-CREB levels in PV-INs from 1-month-old mice (*n* = 6 WT and Tg2576 mice; *p* = 0.223 with Mann–Whitney U test). Confocal images of nuclear *p*-CREB (**B**, red) and c-Fos (**C**, red) levels in CA1 PV-INs (green) of 3-month-old WT and Tg2576 mice. Protein levels of nuclear *p*-CREB (**B**) and c-Fos (**C**) in PV-INs of 3-month-old Tg2576 mice were reduced compared to WT littermates (*p-CREB*: *n* = 5 WT and Tg2576 mice; *p = 0.011 with unpaired *t*-test with Welch’s correction; *c-Fos*: *n* = 5 WT, *n* = 4 Tg2576 mice; ***p = 0.002 with unpaired *t*-test). The nuclei are visualized by DAPI (blue; scale bar: 10 µm each).

Given that the dopaminergic signaling is a key regulator of several pathways converging to CREB phosphorylation [95, 96], regulating neuronal survival and activity, we next investigated if *p*-CREB levels were altered in PV-INs. We analyzed *p*-CREB at 1 and 3 months of age, corresponding to prior and after the onset of VTA dopamine neuron degeneration, respectively [64], that coincide with the ages prior and after the onset of PV-IN loss. We found that nuclear *p*-CREB levels are lower in PV-INs of 3-month-old Tg2576 mice (Fig. 5B), while levels are similar in 1-month-old animals (Fig. 5A). Interestingly, the reduction in *p*-CREB appears to be selective for PV-INs because no changes were found in 3-month-old PNs (Supplementary Fig. 3D).

In line with the fact that *p-*CREB regulates the expression of many early genes containing CRE elements, including c-Fos, we also observed low levels of nuclear c-Fos in PV-INs of 3-month-old Tg2576 mice (Fig. 5C).

Overall, these data prove that the VTA dopamine neuron degeneration leads to an impairment of dopaminergic signaling in PV-INs, associated with reduced *p*-CREB and c-Fos levels that can contribute to the observed hippocampal GABAergic defects.

### Dopaminergic treatments rescue Tg2576 hippocampal network activity

Given that the dopamine drive regulates the function of PV-INs *via* D2 receptors [47, 50, 51], we assumed that the reduction in *p*-CREB and c-Fos is triggered by the reduced activation of D2-like receptors. Thus, we hypothesized that boosting the dopaminergic signaling with D2 receptor agonists could activate the CREB pathway, overall improving PV-IN function.

**Fig. 6:**
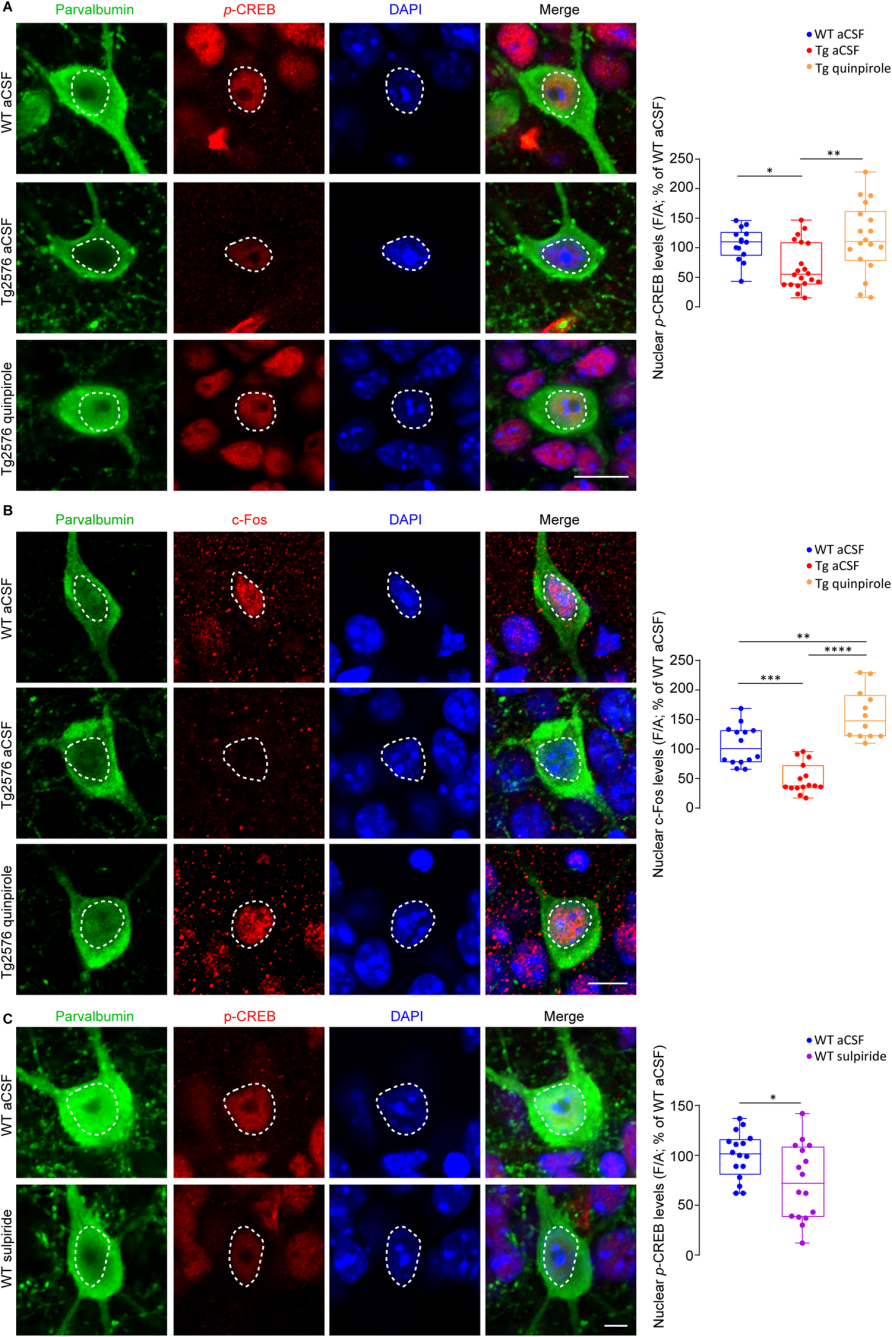
D2-like receptor activation increases *p*-CREB and c-Fos levels in PV-INs of Tg2576 mice. **A** Confocal images of nuclear *p*-CREB (red) in CA1 PV-INs (green; scale bar: 10 µm) from 3-month-old mice in acute slices incubated for 10 min with aCSF or quinpirole 60 µM. Fluorescence intensity levels of nuclear *p*-CREB in PV-INs of 3-month-old Tg2576 aCSF were lower compared to WT aCSF; PV-INs in Tg2576 slices treated with quinpirole show enhanced levels of nuclear *p*-CREB with respect to Tg2576 aCSF (*n* = 14 WT aCSF cells; *n* = 20 Tg2576 aCSF cells; *n* = 18 Tg2576 quinpirole cells; *n* = 3 mice each. One-Way ANOVA interaction *F*_2,49_ = 6.029, *p* = 0.005; Tukey’s post-hoc test: WT aCSF *vs* Tg2576 aCSF: *p = 0.049; Tg2576 aCSF *vs* Tg2576 quinpirole: **p = 0.005). **B** Same as above, showing nuclear c-Fos (red; scale bar: 10 µm). c-Fos nuclear levels in PV-INs of 3-month-old Tg2576 aCSF were reduced compared to WT aCSF; PV-INs of Tg2576 slices treated with quinpirole show high levels of c-Fos with respect to Tg2576 aCSF (F/A % of WT aCSF; *n* = 14 WT aCSF cells; *n* = 15 Tg2576 aCSF cells; *n* = 12 Tg2576 quinpirole; *n* = 3 WT and Tg2576 mice. One-Way ANOVA interaction: *F*_2,38_ = 35.18, *p* < 0.0001; with Tukey’s post-hoc test: WT aCSF *vs* Tg2576 aCSF: ***p = 0.0001; Tg2576 aCSF *vs* Tg2576 quinpirole 60 µM: ****p < 0.0001; WT aCSF *vs* Tg2576 quinpirole 60 µM: **p = 0.0011). The nuclei are visualized by DAPI (blue). **C** Same as above, showing nuclear *p*-CREB levels (red) in CA1 PV-INs (green) from 3-month-old WT mice in acute slices incubated for 10 min with aCSF or sulpiride 100 µM (scale bar: 5 µm). Nuclear *p*-CREB levels in PV-INs of WT sulpiride slices are reduced compared to WT aCSF (*n* = 16 cells/group; *n* = 3 mice; *p = 0.022 with unpaired *t*-test).

We found that in Tg2576 mice the selective D2-like receptor agonist quinpirole increases *p*-CREB and c-Fos levels in PV-INs in 3-month-old hippocampal slices compared to aCSF-treated ones, reaching WT levels (WT aCSF; Fig. 6A, B). To further strengthen this result, we applied the selective D2-like receptor antagonist sulpiride on slices from WT mice, and showed that it reduces *p*-CREB levels in WT PV-INs, mimicking Tg2576 features (Fig. 6C). These data suggest that activity and survival pathways in PV-INs are modulated through the D2-like receptor.

**Fig. 7:**
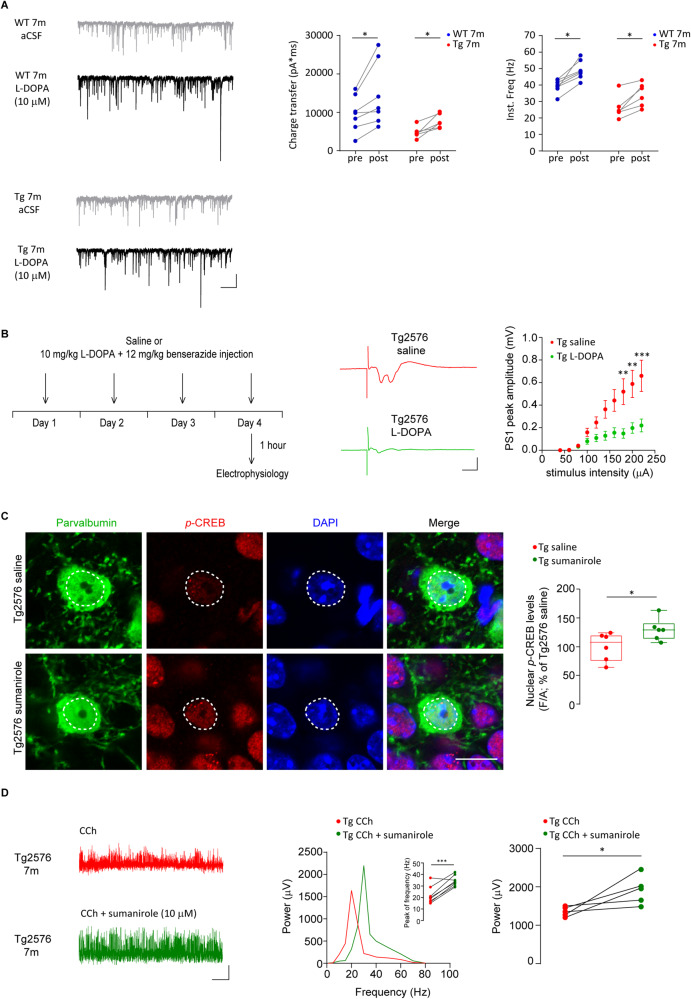
L-DOPA or sumanirole rescues GABAergic activity, reduces hippocampal hyperexcitability and enhances gamma-oscillations in Tg2576 mice. **A** Examples of sIPSCs recorded from WT (*Top*) and Tg2576 (*Bottom*) CA1 PNs at 7 months of age before (*gray*) and after (*black*) bath-applied L-DOPA 10 μM (scale bars: 50 pA; 1 s). Plots show that L-DOPA increases charge transfer (*left*) and  Inst. Freq. (*right*) of sIPSCs in both Tg2576 and WT mice (*n* = 7 cells, 3 WT mice; *n* = 6 cells, 3 Tg2576 mice; WT aCSF *vs* WT L-DOPA: charge transfer *p = 0.016; Inst. Freq: *p = 0.016; Tg2576 aCSF *vs* Tg2576 L-DOPA: charge transfer *p = 0.031; Inst. Freq: *p = 0.031; all with Wilcoxon matched-pairs signed rank test). **B** (*Top*) Experimental procedure for the sub-chronic treatment of saline or L-DOPA (10 mg/kg + benserazide 12 mg/kg) in 7-month-old WT and Tg2576 mice. Animals received single i.p. injections (5 μl/grams of weight) for 4 consecutive days; mice were sacrificed 1 h after the last injection to perform electrophysiological recordings. (*Bottom)* Representative POPs recorded from the CA1 *stratum pyramidale* following half-maximal Schaffer collateral stimulation in 7-month-old Tg2576 mice with sub-chronic L-DOPA or saline treatment (*left*; scale bars: 0.5 mV; 10 ms) and relative I/O curves (*right*) at increasing stimulation. I/O curves show that the peak amplitude of POPs in Tg2576 mice treated with L-DOPA is reduced compared to Tg2576 animals injected with saline (*n* = 10 Tg2576 saline, *n* = 8 Tg2576 L-DOPA slices; *n* = 3 Tg2576 mice; I/O curves: Two-way RM ANOVA with Sidak’s multiple comparisons post-hoc test, treatment × stimulus intensity, *F*_9,153_ = 6.621, *p* < 0.0001; **p = 0.003, 180 μA; **p = 0.002, 200 μA, ***p = 0.0002, 220 μA). **C** Confocal images of nuclear *p*-CREB (red) in CA1 PV-INs (green) of 3-month-old Tg2576 mice injected with sumanirole (5 mg/kg, i.p.) or saline (scale bar: 5 µm). Nuclear *p*-CREB levels in PV-INs from Tg2576 mice injected with sumanirole are increased compared to saline-injected Tg2576 (*n* = 6 mice/group; *p = 0.043 with unpaired *t*-test). **D** Representative traces of CCh-induced local field potentials recorded in the hippocampal pyramidal layer of 7-month-old Tg2576 slices before and during sumanirole bath-application (scale bars: 500 ms, 200 μV). On the left, a representative power spectra plot is shown together with an inset showing the peak of frequency increasing following sumanirole (*n* = 8 slices/group; *n* = 3 Tg2576 mice; ***p = 0.0007 paired *t*-test); on the right, the relative plot indicates that bath-applied sumanirole increases gamma-power in Tg2576 slices (*n* = 3 Tg2576 mice; *n* = 5 slices/group; *p = 0.048 paired *t*-test).

To prove that the increased *p*-CREB and c-Fos levels in Tg2576 PV-INs following the boost of the dopaminergic tone reflect the improvement of GABAergic inhibition in Tg2576 mice, we assessed the acute and sub-chronic effects of L-DOPA. L-DOPA bath-application enhanced the inhibitory drive on PNs by increasing the sIPSC total charge transfer and frequency (Fig. 7A). Importantly, this effect was reproduced by sub-chronic treatment (Fig. 7B) that ameliorates hippocampal hyperexcitability in 7-month-old Tg2576, by reducing the PS1 I/O curve (Fig. 7B) without affecting the PS1 duration, total number and total POP duration (Supplementary Fig. 3E).

Finally, we tested the effects of sumanirole, another highly selective D2-like receptor agonist, demonstrated to be an effective anticonvulsant in experimental models of frontal lobe onset seizures [47]. We asked whether sumanirole could ameliorate the PV-IN-mediated functions in Tg2576 mice. We found that, similarly to the effects of quinpirole, *p*-CREB levels were increased in Tg2576 following the in-vivo administration of sumanirole compared to saline-injected Tg2576 mice (Fig. 7C). Importantly, sumanirole also enhances gamma-oscillations in Tg2576 (Fig. 7D).

Overall, these results show that increasing dopamine levels restores hippocampal GABAergic activity, thus ameliorating gamma-waves and hyperexcitability in AD mice; we further prove that these effects are mediated by the D2-like receptors expressed on hippocampal PV-INs.

## Discussion

Using multiple approaches, applied to the Tg2576 model of AD [78, 84], we report here evidence for aberrant gamma-waves and hippocampal hyperexcitability associated with a weak inhibitory tone on PNs; this is associated with reduced firing activity and lower numbers of PV-INs, as well as decreased density of the surrounding PNNs. We detected these alterations in the Tg2576 hippocampus from 3 months of age, when only slight behavioral alterations are evident, whilst no Aβ-plaques are yet present [97, 98]. Remarkably, the defective GABAergic function we observe in Tg2576 coincides with a reduced hippocampal dopamine outflow, resulting from the progressive degeneration of VTA dopaminergic neurons since 2-3 months of age, as we previously described [63–65]. The dopamine neuron degeneration is confirmed here by the reduced number of dopamine neurons in the VTA and the lower density of dopaminergic fibers in the dorsal hippocampus, and, above all, by the lower number of dopaminergic boutons onto Tg2576 PV-INs.

The immediate effect of dopamine neuron degeneration on survival and activity of Tg2576 PV-INs is here represented by the evidence of lower activation of the CREB/c-Fos pathway specifically in these cells, downstream of D2-like receptors. Consequently, the D2 agonists quinpirole, sumanirole or L-DOPA rescued functional and molecular GABAergic defects – including gamma-waves and hyperexcitability – confirming the key role of the dopaminergic transmission in regulating the hippocampal network integrity. In line with this notion, D2 receptor antagonism by sulpiride reduces the CREB/c-Fos pathway in WT PV-INs, mirroring what occurs in Tg2576.

Overall, the picture emerging from these results demonstrates that the Tg2576 hippocampal hyperexcitability parallels the lowering of the dopaminergic tone following the degeneration of VTA dopamine neurons.

Our data provide a new point of view in understanding the mechanisms driving hippocampal hyperexcitability in AD, going beyond the mere load of Aβ oligomers and plaques. Indeed, many evidence support the hypothesis that brain hyperexcitability is tied to the Aβ burden [7, 8], and propose a detrimental vicious cycle in which Aβ peptides induce neuronal hyperexcitability, and neuronal hyperactivity enhances the brain’s vulnerability to Aβ [19, 99]. Additionally, what is long known is that some neuronal populations are precociously more sensitive to damaging stressors like Aβ toxic species. Indeed, the extraordinary energy demands needed to support the great amount of excitatory and inhibitory inputs received, and the peculiar high-frequency firing crucial for gamma-waves and PN synchronization, make fast-spiking PV-INs extremely vulnerable and prone to loss of mitochondrial membrane integrity, metabolic and oxidative stress, calcium dysregulation, aberrant synaptic inputs and inflammation [93, 100, 101]. To counteract this susceptibility, PV-INs are protected by surrounding PNNs, whose synthesis and degradation rely also on PV-IN firing activity [94, 102]. In this context, our data from Tg2576 mice provide a new insight demonstrating that the reduced hippocampal dopamine input onto PV-INs contributes to their reduced firing, possibly also triggering PNN regression. Moreover, the reduced dopaminergic innervation affects PV-IN survival through the defective activation of the CREB/c-Fos signaling. Thus, the loss of dopamine contributes to the increased vulnerability of PV-INs, in parallel to the detrimental direct and/or indirect effects of Aβ species and neuroinflammation, affecting neuronal homeostasis, activity and survival. Thus, it is not surprising that PV-INs and their PNNs across different AD models – including Tg2576 as shown in this manuscript – undergo early alterations, overall resulting in PV-IN malfunction/degeneration and hippocampal hyperexcitability [101, 103, 104]. Of note, other INs might also be affected by the dopamine loss, for example the somatostatin neurons that also produce slow gamma-oscillations [105].

Moreover, our data show that *f*EPSPs are not changed in Tg2576 slices, in line with earlier observations [64, 66]. Yet, this does not rule out the presence of glutamatergic deficits in these mice. Indeed, both we and others have shown important dysregulations in PN excitability in both the dorsal and ventral hippocampus, together with deficits in synaptic plasticity (LTP/LTD), subiculum-nucleus accumbens (NAc) glutamatergic transmission and AMPA receptor reductions [64, 66, 67, 84, 97]. Interestingly, all these deficits are associated with dopamine loss [63, 64, 66, 67] and are rescued by dopamine treatments [63, 64, 66]. Taken together, our findings demonstrate that the degeneration of VTA dopamine neurons is an early event occurring during the pre-symptomatic AD stage, an event that can explain the behavioral and functional hippocampal changes affecting Tg2576 mice.

The key role of the VTA in the pathophysiology of AD is also corroborated by clinical studies demonstrating that VTA functional, structural and metabolic changes affect the mesocorticolimbic system in aMCI patients, while VTA disconnection with projecting areas speeds up the conversion from MCI to AD-dementia [56, 59, 61]. In parallel, clinical evidence demonstrate altered gamma-oscillations or subclinical epilepsy in MCI/early-AD patients, determining a fast progression of cognitive symptoms [11]. Interestingly, clinical studies have also shown a correlation between the level of gamma activity in the temporal cortex and epileptiform activity, with global connectivity in the gamma frequency band significantly reduced only in MCI patients with epileptiform activity [106]. Yet, the clinical evidence linking subclinical epilepsy with mesocorticolimbic system dysfunctions is still missing. To this aim, it is urging to investigate if aMCI patients, with proven VTA disconnection in mesocorticolimbic targets, are also affected by aberrant gamma-waves, brain hyperexcitability or subclinical epilepsy.

Thus, our work has relevant clinical implications, hinting that the precocious degeneration of the mesocorticolimbic dopamine system leads to neuronal hyperexcitability in AD. In line with this concept, the use of dopaminergic D2 agonists in the management of epilepsy – such as sumanirole used in this study – can be traced back over years [47, 107], but our findings open the way to the use of dopaminergic drugs precociously, to counteract hippocampal hyperexcitability since the MCI stage, in the attempt to slowdown the progression from MCI to clinical AD.

### Supplementary information

Supplementary informations

Supplementary Figure 1

Supplementary Figure 2

Supplementary Figure 3

## Data Availability

All data generated or analyzed during this study are included in this article and its supplementary information files and could be made available upon reasonable request from the corresponding author.
