# Supplementary material for: Dopamine neuron degeneration in the Ventral Tegmental Area causes hippocampal hyperexcitability in experimental Alzheimer’s Disease
Source: Mol Psychiatry. 2024 Jan 16;29(5):1265–80. doi: 10.1038/s41380-024-02408-9 (PMC11189820; doi:10.1038/s41380-024-02408-9)
Supplement: Supplementary file 1 — Supplementary informations [file 41380_2024_2408_MOESM1_ESM.docx]

**Supplementary Information for**

**Dopamine neuron degeneration in the ventral tegmental area causes hippocampal hyperexcitability in experimental Alzheimer’s Disease**

Elena Spoleti^1,†^, Livia La Barbera^1,2,†^, Emma Cauzzi^3^, Maria Luisa De Paolis^1^, Luana Saba^1,2^, Ramona Marino^1^, Giuseppe Sciamanna^2,4^, Vincenzo Di Lazzaro^1,5^, Flavio Keller^1^, Annalisa Nobili^1,2^, Paraskevi Krashia^2,6,*^ and Marcello D’Amelio^1,2,*^

1. Department of Medicine and Surgery, Università Campus Bio-Medico di Roma, 00128 Roma, Italy.

2. Department of Experimental Neurosciences, IRCCS Santa Lucia Foundation, 00143 Rome, Italy.

3. Department of Systems Medicine, University of Rome Tor Vergata, 00133 Rome, Italy.

4. UniCamillus International University of Health Sciences, 0131 Rome, Italy

5. Operative Research Unit of Neurology, Fondazione Policlinico Universitario Campus Bio-Medico, 00128 Rome, Italy

6. Department of Sciences and Technologies for Sustainable Development and One Health, Università Campus Bio-Medico di Roma, 00128, Rome, Italy.

† These authors contributed equally to this work.

* These authors contributed equally to this work.

Correspondence to: Marcello D’Amelio, Department of Medicine and Surgery, Università Campus Bio-Medico di Roma, Via Álvaro del Portillo, 21 00128 Rome, Italy

E-mail: m.damelio@unicampus.it

**Supplementary Content**

- Word file containing Supplementary Table 1, and captions for Supplementary Figure Legends
- Supplementary Fig. 1 in EPS
- Supplementary Fig. 2 in EPS
- Supplementary Fig. 3 in EPS

**Supplementary Table 1**

**Table 1. Electrophysiological properties (mean ± s.e.m) of CA1 PV-INs of 7-month-old mice PV-Cre/WT and PV-Cre/Tg2576 mice**

| **Parameters** | **PV-Cre/WT** | **PV-Cre/Tg2576** | **Statistics** |
| --- | --- | --- | --- |
| V_threshold_ (mV) | -48.28 ± 2.69 (10) | -42.00 ± 4.329 (6) | 0.258 |
| Rheobase (pA) | 93.82 ± 19.15 (10) | 83.33 ± 26.70 (6) | 0.635 |
| SFA | 0.587 ± 0.074 (5) | 0.581 ± 0.041 (6) | 0.792 |
| CV-ISI | 0.174 ± 0.042 (7) | 0.167 ± 0.016 (6) | 0.893 ^a^ |
| C_m_ (pF) | 26.04 ± 1.70 (11) | 27.85 ± 2.34 (6) | 0.541 ^a^ |
| R_m_ (MΩ) | 260.40 ± 15.56 (12) | 252.60 ± 36.21 (6) | 0.846 ^a^ |
| RMP (mV) | -54.74 ± 1.97 (10) | -59.82 ± 3.40 (6) | 0.067 |
| R_in_ (MΩ) | 135.70 ± 9.05 (11) | 105.31 ± 5.83 (6) | 0.122 |

The number of analyzed neurons is shown in brackets. Statistics (for WT vs Tg2576): Mann-Whitney U test except for Welch’s t-test (^a^).

Abbreviations: C_m_ = Membrane Capacitance; CV-ISI = Coefficent of variance – Inter Spike Interval; R_in_ = Input Resistence; R_m_ = Membrane Resistence; RMP = Resting Membrane Potential; SFA = Spike Frequency Adaptation; V_threshold_ = potential threshold

**Supplementary Figure 1: Additional data from recordings of POPs**

**A)** Plots show similar values for analyzed parameters of POPs recorded at half-maximal intensity between genotypes in (*top*) 1-month-old (n=17 slices, 4 WT mice; n=13 slices, 4 Tg2576 mice; PS1 peak amplitude: p=0.133 with Mann-Whitney U test; PS1 duration: p=0.169 with unpaired *t*-test with Welch’s correction; total peak number: p=0.122 with Mann-Whitney U test; POPs total duration: p=0.698 with Mann-Whitney U test), (*middle*) 3-month-old (n=7 slices, 4 WT mice; n=8 slices, 6 Tg2576 mice; PS1 peak amplitude: p=0.295 with Mann-Whitney U test; PS1 duration: p=0.131 with unpaired *t*-test with Welch’s correction; POPS peak total number: p=0.843 with unpaired *t*-test with Welch’s correction; POPS total duration: p=0.282 with unpaired *t*-test with Welch’s correction), and (*bottom*) 7-month-old mice, except for PS1 peak amplitude (n=14 slices, 6 WT mice; n=12 slices, 6 Tg2576 mice; PS1 peak amplitude: *p=0.014 with unpaired *t*-test with Welch’s correction; PS1 duration: p=0.958 with unpaired *t*-test with Welch’s correction; total peak number: p=0.205 with Mann-Whitney U test; POPs total duration: p=0.694 with unpaired Welch’s *t*-test). **B)** Examples of POPs recorded from WT and Tg2576 mice at 3- and 7- months of age in control conditions (aCSF; black) and in presence of 5 μM bath-applied Bicuculline (grey; scale bars: 0.25 mV, 5 ms). Plots (± s.e.m.) show the total number, amplitude and duration of evoked peaks in control condition and in presence of Bicuculline (grey area). (*Top*) No changes were observed between genotypes in 3-month-old animals (n=6 slices, 4 WT mice, n= 8 slices, 6 Tg2576 mice; two-way RM ANOVA with Bonferroni’s multiple comparisons post-hoc test, genotype x stimulus intensity; number of peaks: F_4,48_=2.428, p=0.061; max peak: F_4,48_=0.209, p=0.932; max duration: F_4,48_=1.286, p=0.289). (*Bottom*) The total number and the duration of evoked responses are increased in 7-month-old Tg2576 mice compared to WT littermates; no differences were detectable in terms of peak amplitude (n=9 slices, 4 WT mice; n=11 slices, 6 Tg2576 mice; two-way RM ANOVA with Bonferroni’s multiple comparisons post-hoc test, genotype x stimulus intensity; number of peaks: F_4,72_=2.548, p=0.047; *p=0.028 at 6 min; max peak: F_4,72_=0.151, p=0.208; max duration: F_4,72_=3.578, p=0.010; *p=0.0101 at 6 min; *p=0.029 at 9 min).

**Supplementary Figure 2: Unchanged *f*EPSPs in Tg2576 mice**

Representative *f*EPSPs recorded from the CA1 *stratum radiatum* following half-maximal Schaffer collateral stimulation (scale bars: 0.5 mV; 10 ms) and related I/O curves (± s.e.m.) in 3- and 7-month-old Tg2576 and WT mice; I/O curves are similar between genotypes across ages (two-way RM ANOVA with Bonferroni’s multiple comparisons test, genotype x stimulus intensity; *(left)* 3-month-old: n=7 slices, 4 WT mice; n=9 slices, 6 Tg2576 mice, F_10,140_=0.169, p=0.998; (*right*) 7-month-old: n=14 slices, 6 WT mice; n=11 slices, 6 Tg2576 mice, F_10,230_=1.663, p=0.091).

**Supplementary Figure 3: Additional data of PV-IN soma, cell numbers, and expression of nuclear *p*-CREB, cell counts of VTA DA neurons, and various POP parameters following L-DOPA treatment**

**A)** The plot shows no differences in the PV-IN soma area between genotypes at both ages (*3-month-old*: n=6 WT and Tg2576 mice; p=0.379; *7-month-old*, n=9 WT andTg2576 mice; p=0.190; unpaired *t*-test with Welch’s correction). **B)** The plot indicates similar stereological cell counts for PV^+^/PNN^+^ INs between genotypes in 1-month-old mice (n=10 WT, n=9 Tg2576 mice; PV^+^/PNN^+^ labelling p=0.081; with unpaired *t*-test with Welch’s correction). **C**) The stereological cell count shows reduced number of TH^+^ neurons in the VTA of 7-month-old DAT-Cre/Tg2576 compared to DAT-Cre/WT mice (n=4 WT, n=3 Tg2576 mice; **p=0.004 with unpaired *t*-test). **D)** The plot indicates similar nuclear levels of *p*-CREB in PNs across genotypes at 3 months of age (n=5 WT and Tg2576 mice; p=0.194 with unpaired *t*-test). **E)** Plots show no differences between Tg2576 mice sub-chronically treated with saline or L-DOPA (10 mg/kg + benserazide 12 mg/kg) in terms of PS1 duration, total peak number and total POPs duration recorded at the half-maximum stimulation intensity (n=10 Tg2576 saline, n=8 Tg2576 L-DOPA slices; n=3 Tg2576 mice each; PS1 duration: p=0.914; total peak number: p=0.725; POPs total duration: p=0.813; all with unpaired *t*-test with Welch’s correction).
