# Supplementary figures and images for: Dopamine neuron degeneration in the Ventral Tegmental Area causes hippocampal hyperexcitability in experimental Alzheimer’s Disease
Source: Mol Psychiatry. 2024 Jan 16;29(5):1265–80. doi: 10.1038/s41380-024-02408-9 (PMC11189820; doi:10.1038/s41380-024-02408-9)

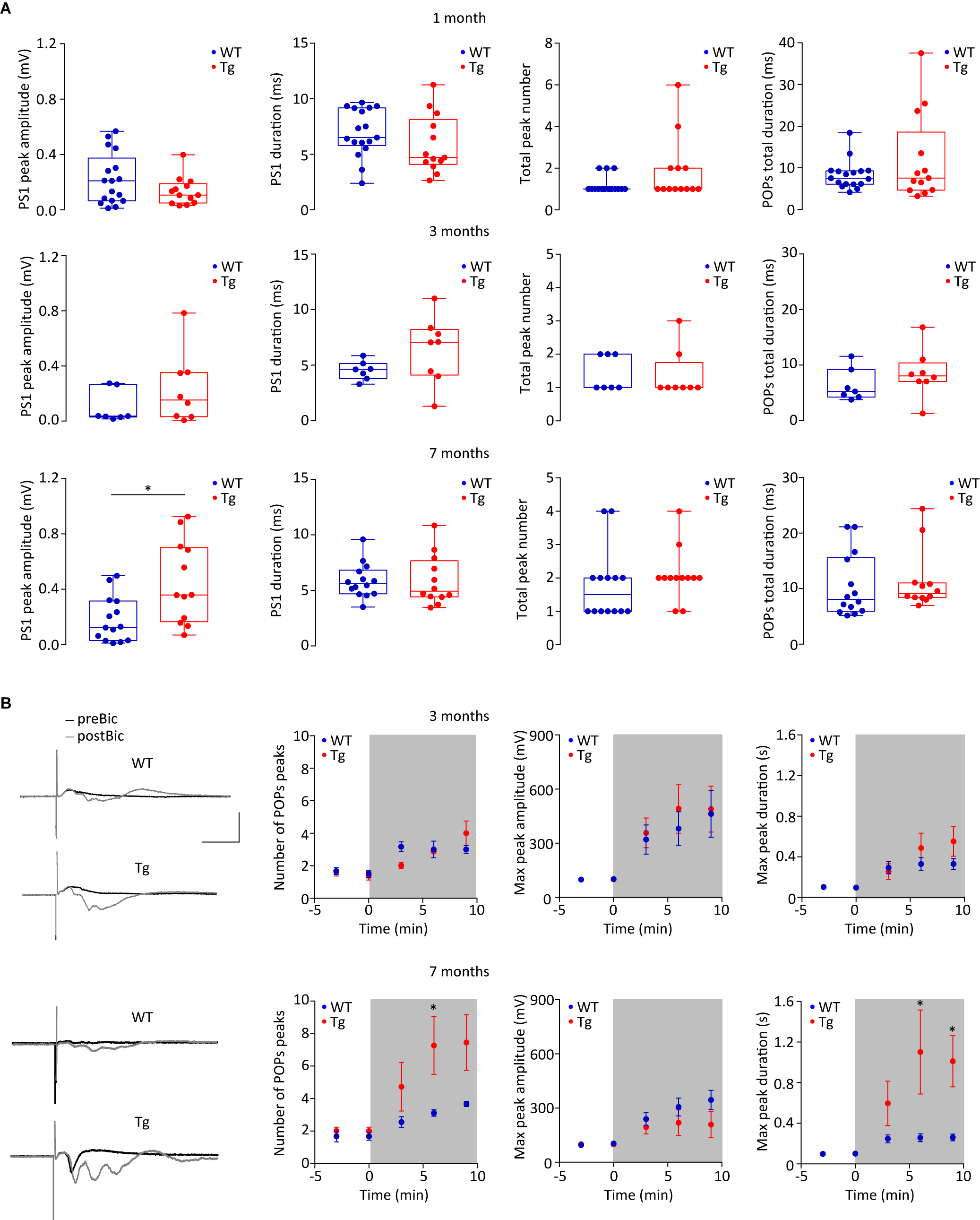

Supplement: Supplementary file 2 — Supplementary Figure 1 [file 41380_2024_2408_MOESM2_ESM.tif]

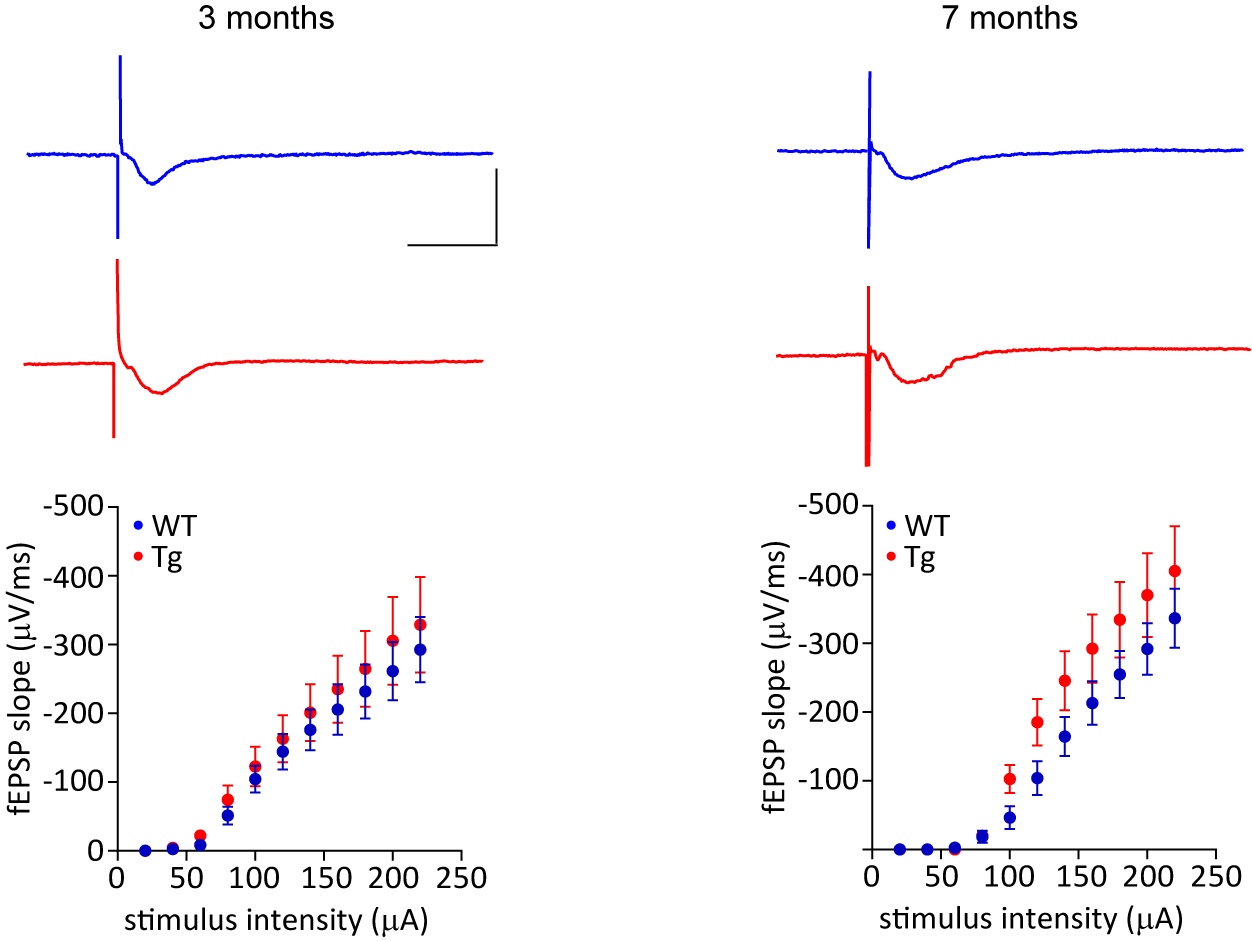

Supplement: Supplementary file 3 — Supplementary Figure 2 [file 41380_2024_2408_MOESM3_ESM.tif]

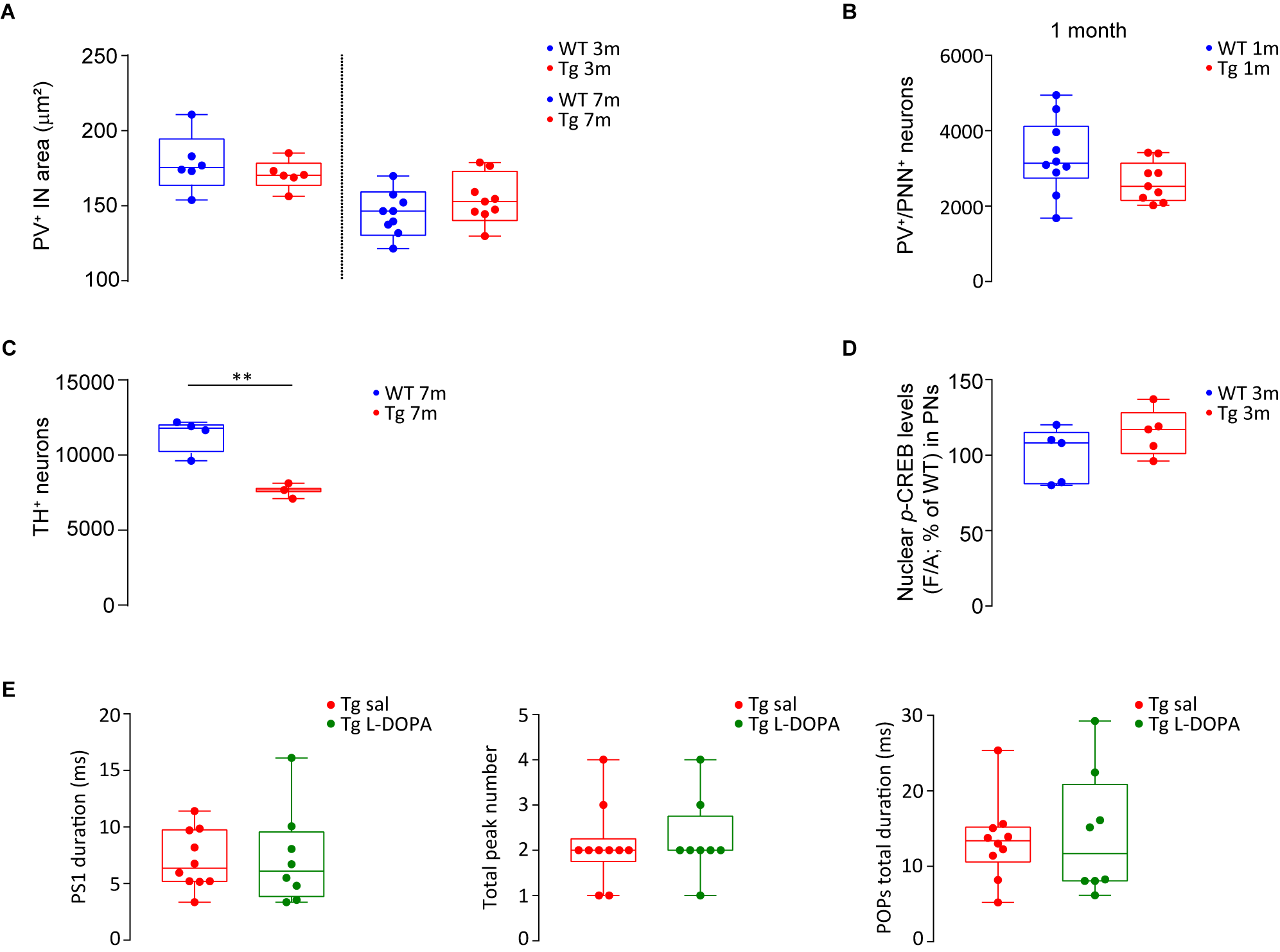

Supplement: Supplementary file 4 — Supplementary Figure 3 [file 41380_2024_2408_MOESM4_ESM.tif]
